# Xylem Anatomical Variability in White Spruce at Treeline Is Largely Driven by Spatial Clustering

**DOI:** 10.3389/fpls.2020.581378

**Published:** 2020-10-21

**Authors:** Timo Pampuch, Alba Anadon-Rosell, Melanie Zacharias, Georg von Arx, Martin Wilmking

**Affiliations:** ^1^Landscape Ecology and Ecosystem Dynamics Working Group, Institute of Botany and Landscape Ecology, University Greifswald, Greifswald, Germany; ^2^General and Special Botany Working Group, Institute of Botany and Landscape Ecology, University Greifswald, Greifswald, Germany; ^3^Swiss Federal Institute for Forest, Snow and Landscape Research WSL, Birmensdorf, Switzerland

**Keywords:** boreal forest, broad-sense heritability, clonal trees, spatial clustering, treeline, white spruce, xylem anatomy

## Abstract

The ecological function of boreal forests is challenged by drastically changing climate conditions. Although an increasing number of studies are investigating how climate change is influencing growth and distribution of boreal tree species, there is a lack of studies examining the potential of these species to genetically adapt or phenotypically adjust. Here, we sampled clonally and non-clonally growing white spruce trees (*Picea glauca* [Moench] Voss) to investigate spatial and genetic effects on tree ring width and on six xylem anatomical traits representing growth, water transport, mechanical support, and wood density. We compared different methods for estimating broad sense heritability (H^2^) of each trait and we evaluated the effects of spatial grouping and genetic grouping on the xylem anatomical traits with linear models. We found that the three different methods used to estimate H^2^ were quite robust, showing overall consistent patterns, while our analyses were unsuccessful at fully separating genetic from spatial effects. By evaluating the effect size, we found a significant effect of genetic grouping in latewood density and earlywood hydraulic diameter. However, evaluating model performances showed that spatial grouping was a better predictor than genetic grouping for variance in earlywood density, earlywood hydraulic diameter and growth. For cell wall thickness neither spatial nor genetic grouping was significant. Our findings imply that (1) the variance in the investigated xylem anatomical traits and growth is mainly influenced by spatial clustering (most probably caused by microhabitat conditions), which (2) makes it rather difficult to estimate the heritability of these traits in naturally grown trees *in situ*. Yet, (3) latewood density and earlywood hydraulic diameter qualified for further analysis on the genetic background of xylem traits and (4) cell wall thickness seems a useful trait to investigate large-scale climatic effects, decoupled from microclimatic, edaphic and genetic influences.

## Introduction

Boreal forests are ecologically and commercially valuable ecosystems that make up almost a third of the global forest cover (Apps et al., 1993; Hansen et al., 2003). They act as a sink for global atmospheric carbon dioxide (Arneth et al., 2010; Tagesson et al., 2020) but are heavily influenced by human-induced and natural cover loss (Hansen et al., 2010). According to climate projections, boreal forests will face exceptional changes in climatic conditions within the 21st century (Soja et al., 2007; IPCC, 2013; Charney et al., 2016), threatening ecosystem functions (Gauthier et al., 2015). To preserve their functionality, it is of outmost importance to understand how these ecosystems work and how boreal forest tree species adjust to environmental changes.

A great majority of tree species are able to cope with a range of environmental conditions (Reich et al., 2016). To this end, they adjust phenotypically, adapt genetically and/or disperse into new habitats to track their niche of suitable conditions (Lenoir et al., 2008; Yeaman et al., 2016). In sessile and long-lived organisms like trees, the ability to adjust to changing conditions is essential (Schlichting, 1986). However, in the long term it is also necessary for a population to adapt genetically. Adaptation can occur when phenotypes of traits improving fitness are heritable. A common way to quantify heritability of a trait is to estimate the amount of phenotypic variance of the trait that occurs due to genetic variance. Heritability can be estimated in a “narrow sense” (h^2^, based on additive genetic variance) and a “broad sense” (H^2^, based on total genetic variance; Visscher et al., 2008; Wray and Visscher, 2008). Estimating heritability of traits in trees can thus help to inform species distribution models to create more precise predictions of future development of forests, and can also guide projects aiming at maintaining the functionality of boreal forests (e.g., assisted migration; Gauthier et al., 2015; Correia et al., 2018).

White spruce (*Picea glauca* [Moench] Voss) is one of the most common tree species of the North American boreal forests (Little and Viereck, 1971). Due to its ability to grow at the latitudinal and altitudinal treeline, it is widely used as a model organism to study plasticity and adaptation patterns (Lloyd and Fastie, 2002; Wilmking and Juday, 2005; Sherriff et al., 2017). Most studies on this species focus on general tree growth, often exclusively investigating annual (radial) growth increments. While tree rings provide valuable information on the integrated response to environmental conditions during the vegetation period, investigating the xylem anatomical structure may reveal crucial information on the tree functionality (Hacke et al., 2015; Amoroso et al., 2017).

Studies investigating xylem anatomical traits that are directly related to tree functioning such as tracheid lumen diameter or cell wall thickness (Wiedenhoeft, 2012) have become increasingly available for boreal tree species (Lange et al., 2019; Mvolo et al., 2019). Yet, little is known about the genetic background of xylem anatomical trait variation in white spruce (Lenz et al., 2010; Hassegawa et al., 2019). White spruce is able to vegetatively reproduce by layering (Stone and McKittrick, 1976; Würth et al., 2018) and thus it is able to grow genetically identical individuals (i.e., clones). Clones offer the unique opportunity to study genetic effects (i.e., broad sense heritability) on growth, hydraulic and structural traits in natural populations (Nyquist and Baker, 1991).

In this study, we identified and sampled naturally growing clones of white spruce at the latitudinal treeline in Alaska. We aimed at estimating broad sense heritability (H^2^) of growth and xylem anatomical traits of three trait groups (water transport, mechanical support, and wood density) by comparing three different methods: (1) using raw data, (2) using data predicted with a linear mixed effects model and (3) using estimated variance extracted from a linear mixed effects model. Since vegetative reproduction in trees leads to an unavoidable spatial clustering of individuals, we additionally focused on the spatial patterns. We evaluated the results of our H^2^ estimations by using models to identify whether spatial grouping is the main driver for similarities in growth and xylem anatomical traits, or if genetics also influence these patterns. The advantage of this novel approach is that it combines spatial analyses with genetic analyses at an anatomical level. This informs about which xylem anatomical traits qualify for studying genetic patterns potentially leading to genetic adaptation and which qualify better for studying spatial patterns driven by the influence of microenvironment or by climatic effects.

## Materials and Methods

### Study Species and Site

White spruce grows under a variety of climatic conditions and its distributional range covers most of the boreal area in Canada and Alaska, and parts of the northernmost United States mainland (Little and Viereck, 1971). It is often the dominant tree species at the elevational and latitudinal treeline in the north-western parts of its distributional range (Abrahamson, 2015), and is of large economic importance (Attree et al., 1991).

The study site is located at the latitudinal treeline on a south-facing slope of Nutirwik Creek valley, in the central Brooks Range of Alaska (67°56′N, 149°44′W). The study site is a nearly monospecific white spruce stand of approximately two hectares, ranging in elevation from 860 to 940 m a.s.l. The mean annual temperature is around −7.9°C with a mean temperature of around −23.8°C in January (coldest month) and 11.1°C in July (warmest month). The annual precipitation is around 289 mm, 96 mm of which fall in July and August. The information about precipitation and temperature is taken from Lange et al. (2019) and based on modeled data averaged across the 1901–2013 data period provided by the Natural Resources Canada, Canadian Forest Service (NRCAN^1^; McKenney et al., 2011).

### Sampling Design and Data Acquisition

**FIGURE 1:**
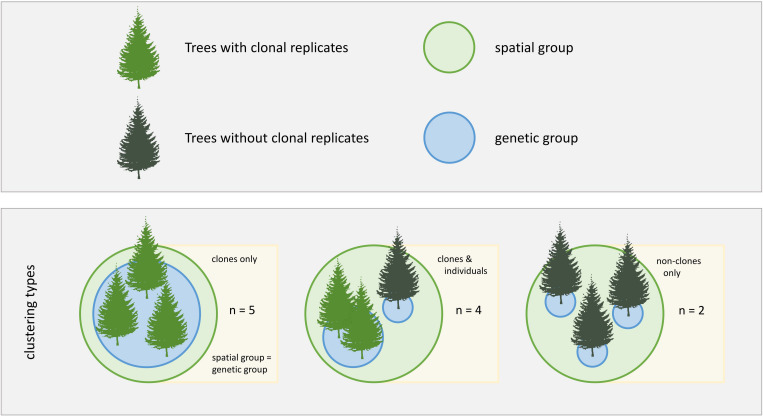
Study design. Different clonal contexts and grouping types are indicated by differently colored trees and circles, respectively. Three clustering types were analyzed in this study, with *n* being the number of study cases for each clustering type.

In 2018, we sampled all the spatially clustered groups of white spruce that were scattered throughout the study area ranging from the forest line to the treeline and appearing to be the result of vegetative reproduction (see Wilmking et al., 2017; Würth et al., 2018) (Figure 1). We sampled all trees present within each spatially clustered group (Supplementary Figure S1 and Supplementary Table S1). We took one bark-to-bark increment core through the pith from 47 trees (thus resulting in 94 radii) that grew in eleven spatially clustered groups with a 4.3 mm increment borer (Haglöf, Sweden) for growth and xylem anatomical measurements. Additionally, we collected the most recently grown needles from North-facing branches of each tree for genetic analyses.

All cores were air dried and glued onto wooden sample holders. The surface was polished with progressively finer sandpaper (up to 800 grit) and scanned with a flatbed scanner (Epson Perfection V700 Photo; Seiko Epson Corporation, Japan) with 3200 dpi. Ring widths (TRW) were subsequently measured using CooRecorder (version 9.3.1; Cybis Elektronik and Data AB, Sweden) and all radii were cross-dated using CDendro (version 9.3.1; Cybis Elektronik and Data AB, Sweden). We used the cross-dated tree ring chronologies solely to correctly date xylem anatomical measurements. For the analysis, we used TRW measurements obtained from the anatomical sections together with the other anatomical traits.

For the xylem anatomical measurements we cut 12 μm-thick cross-sections from one radius of each tree using a rotary microtome (Leica RM 2245; Leica Camera AG, Germany). The cross-sections were stained with 1:1 safranin and astra blue solution, rinsed with ethanol solutions of increasing concentration (50%, 70%, 96%), mounted on microscope slides with Euparal and dried at 60°C for 48 h. We scanned the slides with a slide scanner (Zeiss Axio Scan.Z1; Carl Zeiss AG, Germany) at the Swiss Federal Institute for Forest, Snow and Landscape Research (WSL), Birmensdorf, Switzerland. We used the scans to quantify TRW and several xylem anatomical traits (Supplementary Table S2) with the image analysis tool ROXAS v3.0.326 (von Arx and Carrer, 2014; Prendin et al., 2017). Due to the large amount of anatomical data to process in long cores, we selected the growth years 2007–2017 for the measurements of xylem anatomical traits. This allowed us to maximize the amount of data while still maintaining a number of samples feasible to process with high quality. Measurements on lumen diameter and cell wall thickness were used to distinguish between early- and latewood using Mork’s index (Denne, 1989). We calculated the total mean, the mean for earlywood and the mean for latewood per year of each trait using R v3.6.1 (R Core Team, 2019).

Earlywood ring width (EWW) and latewood ring width (LWW) were detrended for each tree to minimize the influence of low frequency growth trends without losing too much information on the variance among the trees. For this, we compared two linear models with raw ring width (EWW and LWW separately) as a response variable and year as an explanatory variable. One model was fitted with a linear term of the explanatory variable while the other model was fitted with a linear and a quadratic term of the explanatory variable. We chose the better performing model based on the corrected Akaike Information Criterion (AICc; Hurvich and Tsai, 1991) and calculated the detrended EWW and LWW by adding the model residuals to the mean annual growth of the investigated time period. Mean hydraulic diameter (DH) was calculated for earlywood (DH.ew) and latewood (DH.lw) of each ring based on lumen area (LA) according to Kolb and Sperry (1999). We estimated wood density (DEN) as the proportion of the estimated cell wall area (CWA) to the total cell area [i.e., the sum of CWA and lumen area (LA); Eq. 1] according to Björklund et al. (2017).

(1) $$D\,E\,N=\frac{C\,W\,A}{C\,W\,A+L\,A}$$

### DNA Isolation and SSR Genotyping

To identify clones, we genotyped all sampled trees. We ground 20 mg of silica-gel dried needle tissue in a Retsch ball mill MM301 (Retsch, Germany). For DNA extraction we used the Mag-Bind plant DNA DS Kit (Omega, United States) in combination with the KingFisher^TM^ Flex 96-well plate robot system (ThermoFisher Scientific, United States) following the manufacturer protocols. For genotyping we combined 11 microsatellite loci developed by Hodgetts et al. (2001) and Rajora et al. (2001) in two multiplex reactions according to Eusemann et al. (2014) and Würth et al. (2018). We performed PCR on Eppendorf Mastercyclers (Eppendorf, Germany) using the Qiagen Multiplex PCR Plus Kit (Qiagen, Netherlands) and a modified protocol with a total volume of 10 μl and PCR conditions as described in Würth et al. (2018) with initially 5 min/95°C, 30 cycles 30 s/95°C, 90 s/58°C, 90 s/72°C, final extension 10 min/68°C. For fragment analysis we used a 3130xl Genetic Analyzer (Life Technologies, United States) using 1 μl undiluted PCR product, 0.15 μl 500 GeneScan LIZ^®^ size standard (Life Technologies) and 12 μl HiDi Formamide (Life Technologies).

We performed fragment size determination and binning with the GeneMapper^®^ Software 5.0 (Life Technologies). To account for genotyping errors, we used the algorithm programmed by Schnittler and Eusemann (2010). Since genotyping errors are much more likely to split than to merge clones, we set the threshold for members of a clone to maximum two deviating loci. For the analysis, we considered only trees with a maximum of two null allele-containing loci. These settings are consistent with Würth et al. (2018).

Of the 47 sampled trees in 11 spatially clustered groups, we found that 35 trees belonged to nine clonal groups, while 12 trees did not belong to any clonal group. Five spatially clustered groups consisted of clonal trees only. In four groups, clones grew spatially clustered with non-clonal individuals. Two groups consisted of non-clonal individuals only (Figure 1).

### Statistical Analysis

To reduce the number of study parameters, we explored the relationship between the measured anatomical traits with a principal component analysis (PCA) using the R function prcomp (Grey et al., 1981) (Supplementary Figure S2). We classified the traits in four groups according to the PCA: growth, water transport, mechanical support, and wood density. We chose one representative trait of each group for which we carried out the analyses for earlywood and latewood separately (Table 1).

**TABLE 1:** Explanation of growth and xylem anatomical traits selected for analysis and their ecological function.

**Group**	**Selected traits**	**Unit**	**Explanation**
Growth	EWW, LWW	μm	Earlywood width, latewood width
Mechanical support	CWT.ew, CWT.lw	μm	Mean overall cell wall thickness (earlywood, latewood)
Wood density	DEN.ew, DEN.lw	Proportion	Mean relative anatomical wood density (earlywood, latewood)
Water transport	DH.ew, DH.lw	μm	Mean hydraulic diameter (earlywood, latewood)

The first principle component (PC1) of the PCA explained 41.3% of variance, the second principle component (PC2) explained 28.7% and the third principle component (PC3) explained an additional 14.7% of variance (Supplementary Figure S2). The PCA showed a strong relationship among the traits within each group. Traits associated with mechanical support were mostly explained by PC1 while growth and water transport related traits were mainly explained by PC2. Wood density traits were explained by both PC1 and PC2 to a similar extent. Some latewood traits (e.g., latewood density (DEN.lw) and LWW) were mainly explained by PC3. In the following analyses, we used EWW and LWW as proxies for growth, DH.ew and DH.lw for water transport, CWT.ew and CWT.lw for mechanical support and earlywood density (DEN.ew) and DEN.lw for wood density (Table 1).

For the H^2^-estimations we only used data from trees that were growing in groups of genetically identical individuals (i.e., a subset of 35 trees in nine groups). In order to explore the potential of using estimated data compared to raw data, we used three different methods to estimate H^2^. First (1), we estimated H^2^ using raw data of all selected xylem anatomical traits (H^2^_raw_; Table 1; Eq. 2; For additional information see Eq. S1 – Eq. S5; Klug et al., 2006). Second (2), we fitted a linear mixed effects model for each trait using the nlme package (Pinheiro et al., 2020), where the investigated trait was included as the response variable, clonal group and year were included as fixed effects, cumulative stem diameter at breast height (cDBH) was included as a covariate and tree ID as a random effect. To correct for autocorrelation in time between multiple measurements in each individual, a first-order autoregressive correlation structure was included in the model using the constructor corAR1 of the nlme R package. The Constant Variance Function (varIdent) of the same package was used to account for the non-homoscedastic distribution of residuals between the clonal groups. Since tree height has a strong influence on xylem anatomical traits (Carrer et al., 2015), we used the model to predict trait values on a new set of data where the cDBH (as a proxy for height) was standardized to represent the average growth of the sampled trees in the investigated time period (i.e., an increase of DBH from 11 to 12 cm during the 10-year period resulted in an average annual growth of 0.1 cm). Heritability was then estimated on the predicted values (H^2^_pred_; Eq. 2; Eq. S1 – Eq. S5). Finally (3), we fitted a linear mixed effects model, where the investigated trait was the response variable, year was included as a fixed effect, cDBH as a covariate and clonal group as a random effect. We used the VarCorr function of the nlme R package to extract estimated genetic variance ($\sigma_{\text{G}}^{2}$) and the residual variance ($\sigma_{\text{R}}^{2}$) from the model. The modeled variance was then used to estimate H^2^ according to Eq. 3 (H^2^_mod_). All models were fitted using restricted maximum likelihood estimation (REML).

(2) $$H^{2}=\frac{\sigma_{G}^{2}}{\sigma_{P}^{2}}$$

where $\sigma_{\text{G}}^{2}$ is the genetic variance and $\sigma_{\text{P}}^{2}$ the total phenotypic variance.

(3) $$H^{2}=\frac{\sigma_{G}^{2}}{\sigma_{G}^{2}+\sigma_{R}^{2}}$$

where $\sigma_{\text{R}}^{2}$ is the residual variance extracted from the models.

The coefficient of variation (CV) was calculated as an error measurement for H^2^ estimations (Eq. 4; Everitt, 1999).

(4) $$C\,V=\frac{\sqrt{\sigma_{G}^{2}}}{\bar{x}}$$

where $\sigma_{\text{G}}^{2}$is the genetic variance and $\bar{x}$ the trait total mean.

To evaluate whether the calculated H^2^ values truly represent genetic effects or rather a spatial pattern caused by the spatial grouping of the clonal trees we used the full dataset of 47 sampled trees in 11 spatially clustered groups, including clonal and non-clonal individuals. We created two categorical groups, genetic group and spatial group, and each tree was assigned a level in each. For the genetic group, each tree was assigned either the clonal group ID or, in non-clonal trees, the individual tree ID; for the spatial group, all trees growing spatially clustered, with a maximum distance of 3 m, were assigned the same code, regardless of the genetic background (Figure 1). This method did not allow us to isolate genetic grouping from the spatial grouping, but allowed us to test whether spatial clustering had a large effect on the variability of our study traits. To avoid computational errors related to this issue we did not compare spatial and genetic grouping in one model, but we compared three different models for each trait: (i) a null model, (ii) a genetic model and (iii) a spatial model. The (i) null model was fitted using the selected trait as the response variable, year as a fixed effect, cDBH as a covariate and tree ID as a random effect. The (ii) genetic model was fitted with the genetic group and year as fixed effects, cDBH as a covariate and tree ID as a random effect. The (iii) spatial model was fitted with the spatial group and year as fixed effects, cDBH as a covariate and tree ID as a random effect. The corAR1 constructor was used in all three models to account for autocorrelation in time within tree individuals. The varIdent function was used in the spatial and genetic models to account for non-homoscedastic distribution of the residuals between groups. All models were fitted using REML.

For the comparison of the model performance we calculated the corrected Akaike Information Criterion (AICc) for each model and selected the model that performed best when the AICc was lowest with more than four units difference. Since AICc is only slightly penalizing small differences in the number of parameters, we considered the null model best in case of equal values, following the principle of parsimony (Burnham and Anderson, 2002, 2004).

To evaluate the effect of spatial and genetic grouping and to find potential significant effects of spatial or genetic clustering that are independent of model performance we conducted an analysis of variance (ANOVA; Chambers and Hastie, 1992). We used R v 3.6.1 (R Core Team, 2019) for all statistical analyses.

## Results

### H^2^ Estimates of Growth and Xylem Anatomical Traits

**FIGURE 2:**
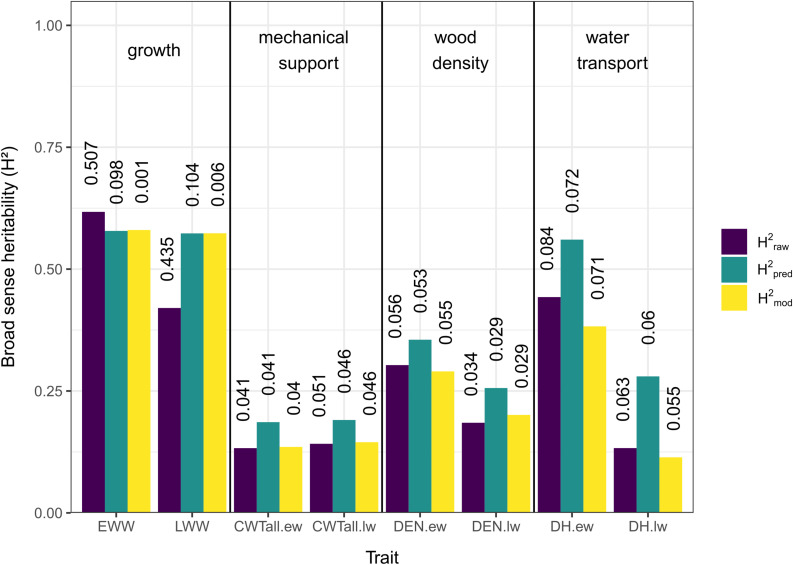
Broad sense heritability (H^2^) estimates for growth and xylem anatomical traits. Different colors indicate the method used (purple = heritability based on raw measurements, green = heritability based on predicted values, yellow = heritability based on mixed-effect model), see “Materials and Methods” section for details. Vertical numbers above the columns are the coefficients of variation (CV).

The three different methods used to estimate H^2^ showed overall similar results (Figure 2). In general, traits associated with growth (EWW and LWW) showed the highest H^2^ values and traits associated with mechanical support (CWT.ew and CWT.lw) the lowest. Traits associated with wood density and water transport (DEN.ew, DEN.lw, DH.ew, and DH.lw) showed low to intermediate values (Figure 2).

In traits related to growth, H^2^_pred_ and H^2^_mod_ showed a pattern of similar values, while H^2^_raw_ was slightly higher for EWW and lower for LWW. For traits related to mechanical support, wood density, and water transport, H^2^_pred_ showed the highest values, while H^2^_raw_ and H^2^_mod_ showed similar values both for earlywood and latewood.

Comparing earlywood and latewood, H^2^ values were similar for growth and mechanical support across methods, while earlywood H^2^ values were generally slightly higher for wood density and notably higher for water transport.

The CV values for H^2^_raw_ in both EWW and LWW were notably high (0.507 and 0.435), but for all other estimates and traits they were relatively low (0.001–0.104, mean = 0.052; Figure 2).

### Comparing Genetic and Spatial Grouping

In general, spatial models outperformed all other models. In one case the null model performed better and in two cases genetic and spatial models performed similarly but better than the null model. In no case did the genetic model outperform the other model types.

**FIGURE 3:**
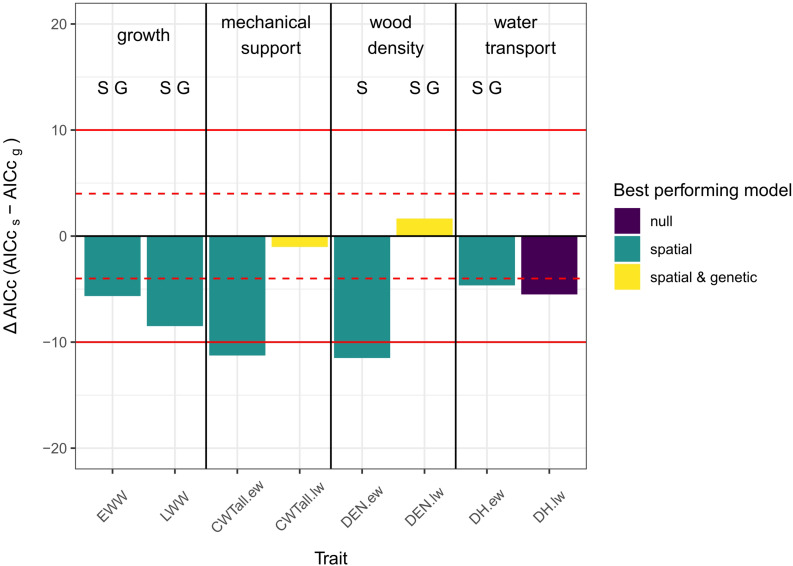
Column plot of the differences in AICc values between spatial and genetic models. A negative ΔAICc means that the AICc of the spatial model is lower than the AICc of the genetic model and vice versa. The color of the columns indicates, which model showed the best performance [purple = null model performed either best, equal to one or equal to both of the other two models; green = spatial model performed best; yellow = grouped models (i.e., genetic and spatial models did not differ significantly between each other but both performed better than the null-model)]. The letters over the columns indicate which model had a significant group effect (S, spatial grouping sign.; G, genetic grouping sign.; significance threshold: *p* < 0.05 - based on ANOVA). Red lines show the thresholds for evaluating differences between the models: |ΔAICc| < 4: no substantial differences (dashed lines); 4 < |ΔAICc| < 10: considerable differences (solid lines); |ΔAICc| > 10 - essential differences (Burnham and Anderson, 2002).

For both growth traits (EWW, LWW) and all earlywood traits (CWT.ew, DEN.ew, and DH.ew) the spatial model performed better than the other models. For latewood density and latewood CWT there was no difference between the genetic and the spatial model, but in both cases the grouped models performed better than the null model. For latewood DH the null model was considered to show the best performance, as it had an AICc equal to the spatial model and lower than the genetic model (Figure 3).

The analysis of the spatial and genetic models using ANOVA showed that the spatial grouping was significant (*p*-value < 0.05) for EWW and LWW, early- and latewood density and earlywood DH. Genetic grouping was significant for EWW and LWW, latewood density and earlywood DH. No significant grouping effect was found in early- and latewood CWT and latewood DH.

## Discussion

The rapidly changing climate conditions threaten the functioning of boreal forests. White spruce is one of the most important and abundant boreal forest tree species of North America. In order to learn more about its potential to adapt to changing environments, we explored its genetic background of xylem anatomical trait variation. The goal of our study was to investigate the broad sense heritability (H^2^) of growth and xylem anatomical traits. We compared three methods to estimate H^2^, which overall showed consistent patterns in the resulting values. However, further analyses revealed a large influence of spatial clustering on xylem anatomy, which seemed to overlay any genetic patterns. Nonetheless, we found some evidence for a genetic influence on early- and latewood growth (ring widths), latewood density and earlywood hydraulic diameter.

### H^2^ Estimates of Growth and Xylem Anatomical Traits

The three methods of estimating H^2^ showed overall consistent results, with the only inconsistency found in the estimates based on raw data (H^2^_raw_) of early- and latewood ring width. This inconsistency was likely caused by a high dispersion in the raw data, shown by high CVs (Figure 2). The H^2^ estimates for EWW and LWW based on the raw data are therefore likely not accurate (Everitt, 1999). All other H^2^ estimates showed much lower CVs and more stable patterns. The H^2^ estimates based on predicted data were higher in most cases. This pattern was likely introduced by the method of using predictions from a linear mixed effects model. By predicting new values under the assumption of equal diameters in all trees we avoided size-related effects, which are known to largely influence xylem anatomical traits (Carrer et al., 2015). The higher values of H^2^_pred_ could suggest an underestimation of H^2^_raw_ and H^2^_mod_ as a result of this size effect.

In general, most of our calculated heritability estimates are in line with other studies focusing on narrow sense heritability (h^2^) of xylem anatomical traits in white spruce. Though narrow sense heritability is based on additive genetic variance instead of total genetic variance, h^2^ is comparable to H^2^ because over 50% of the total genetic variance is usually additive (Hill et al., 2008; Wang et al., 2013). Similar to our study, Lenz et al. (2010) reported a high h^2^ in earlywood radial cell diameter (a trait that strongly correlates with mean hydraulic diameter; Kolb and Sperry, 1999) and a lower h^2^ in the latewood radial cell diameter in white spruce on a provenance trial in East Canada. They also found a low h^2^ in latewood density and latewood cell wall thickness. In contrast to our estimates, Lenz et al. (2010, 2011) reported a high h^2^ in the earlywood cell wall thickness and earlywood density. These opposite results could be explained by their populations being less climatically constrained than our populations. The studies of Lenz et al. (2010, 2011) were conducted on sites in Eastern Canada (Quebec), where climatic conditions are milder and wetter than at our treeline site in northern Alaska. It is possible that the lower heritability estimates in our study are a result of a larger climate control on earlywood parameters at the treeline.

Regarding growth-related traits like ring width, estimates of heritability in white spruce are quite scarce in other studies and considerably differ from our results. While Ying and Morgenstern (1979) and Lenz et al. (2010) reported low, or insignificant levels of h^2^ in DBH (0.04–0.10) and ring width (reported as insignificant), respectively, Corriveau et al. (1991) and Merrill and Mohn (1985) estimated intermediate values for ring width (0.32) and DBH (0.35), respectively. This inconsistency in results might occur due to the complex nature of secondary growth itself (Rathgeber et al., 2016) and the strong influence of climatic parameters (Hughes et al., 2011).

In general, we cannot accurately say how representative our H^2^ estimates are. Per definition, heritability can only be calculated for a specific population in a specific environment (Stoltenberg, 1997). However, the analysis of spatial grouping showed that we have strong spatial effects in our data, which implies that the assumption of common environmental conditions for heritability estimations was violated and thus makes our estimates uncertain.

### Spatial Grouping Has the Strongest Effect on Trait Variability

Comparing models with genetic and spatial grouping showed that in all earlywood traits the spatial model performed better than the genetic model. In the latewood traits, the spatial model was only better for latewood width, but the genetic model did not outperform the spatial model for any trait.

Earlywood is formed at the beginning of the vegetation period. During this time, trees ideally allocate most of the available resources to grow in circumference and height, without risking losing structural integrity or suffering from drought-induced cavitation and other potential effects caused by resource limitation (Willson and Jackson, 2006; Rathgeber et al., 2016; Cartenì et al., 2018). Thus, a high plasticity in the earlywood could promote efficient growth. This high plasticity is evidenced in our results in the form of spatially structured patterns in earlywood anatomical traits. These spatial patterns are probably caused by small-scaled differences in resource availability (i.e., microclimatic and edaphic differences, which are potentially caused by topographic characteristics of the area) between clonal groups, leading to trait variability independent of the genetic background.

At the end of the vegetation period, when latewood is formed, height growth also declines. It becomes more important for the tree to use available resources to produce cells with thicker cell walls, which are responsible for mechanical support for the tree body (Cartenì et al., 2018), while building new tissue for water transport only plays a minor role (Domec, 2002; Tyree and Zimmermann, 2002). Accordingly, latewood anatomy features were less variable in our study. Small-scale differences in resource availability are more likely to cause differences in wood growth and wood density than in hydraulic diameter at the end of the growing season. Consequently, our results showed that for latewood ring width the spatial model was performing best. For the latewood density, genetics might also have an influence, since both models were performing equally well.

Investigating the significance of spatial and genetic grouping independent from the performance of the models revealed a significant effect of genetic grouping on early- and latewood width, latewood density and earlywood DH. Despite the high heritability estimates for early- and latewood width, the effect of genetic grouping was rather unexpected. As mentioned before, secondary growth is strongly influenced by climatic parameters, and several studies suggested that small-scale environmental conditions rather than genetics affect growth (King et al., 2013; Wilmking et al., 2017; Avanzi et al., 2018). Since our study was performed *in situ* we were not able to truly decouple genetic from spatial effects. Therefore, a combination of the strong impact of micro-environmental conditions (i.e., spatial grouping; Avanzi et al., 2018; Montpellier et al., 2018), combined with individual growth characteristics (Carrer, 2011) could have led to the false assumption of a significant genetic effect. We cannot exclude that this also affected the traits latewood density and earlywood DH. However, previous studies already indicated that variability in lumen size and wood density were linked to adaptation to local conditions (Carlquist, 1980; Aroca, 2012; Hacke et al., 2015; Klisz et al., 2019). Thus, it is likely that a genetic effect on latewood density and earlywood DH actually exists.

Both early- and latewood CWT were not significantly influenced by spatial or genetic grouping, indicating that in our study species at our site CWT is not strongly determined by neither small-scale environmental nor genetic effects. We did not test correlations with climatic parameters since our time series (2007–2017) are very short. However, other studies found significant correlations between CWT and climatic parameters in white spruce (Lange et al., 2019) and also in black spruce (Puchi et al., 2020). Placing our results in the context of these studies suggests that CWT qualifies as a proxy for climatic conditions at larger scales (i.e., range-wide differences and past climatic variability), decoupled from strong small-scale environmental and genetic influences. This view is also supported by a study with Scots pine showing that the thickness of the radial cell walls in the latewood registers a stronger temperature signal than any other tree-ring proxy including the commonly used maximum latewood density (Björklund et al., 2020).

## Conclusion

The vegetative reproduction of white spruce at the latitudinal treeline offered the opportunity to gather data on genetically identical trees *in situ*. The comparison of three methods to estimate broad sense heritability (H^2^) resulted in mostly consistent patterns. This suggests that in general the estimates are quite robust, independent from the method used for their calculation. However, due to spatial clustering of the trees we had to evaluate our heritability measures by testing the strength of the grouping effect. The analyses showed that spatial clustering had a strong influence on the xylem anatomy, especially in the earlywood. We assume that this strong spatial effect is related to differences in micro-environmental conditions, which implies that it is rather difficult to estimate the magnitude of genetic effects in a naturally grown population.

Nonetheless, we found some evidence for genetic effects in early- and latewood ring width, latewood density and earlywood hydraulic diameter. Based on previous studies we assume that results on early- and latewood width might rather be reflecting environmental conditions and individual growth patterns than actual genetics. Latewood density and earlywood hydraulic diameter, however, show a plausible significant genetic component, suggesting they are suitable traits for assessing potential local adaptation. Cell wall thickness, on the other hand, seems neither to be influenced by small-scale spatial (i.e., differences that occur within one study site) nor genetic patterns, potentially qualifying as a proxy for climatic conditions on a larger scale (e.g., range-wide differences).

Exploring the interacting effects of phenotypic plasticity and genetic adaptation in xylem anatomical traits related to wood density and tree hydraulics will lead to a more comprehensive understanding of the adaptation potential of tree species to global change in general, and of white spruce in particular. Yet, it is challenging to balance the reliability of experimental set-ups, which may be far from real-life conditions, with real-world studies, which may have high error potential. We believe that real-world studies dealing with clonal trees are valuable, but highlight the necessity to carefully evaluate any potential spatial effects, as they can drastically influence the growth of trees and obscure any potential genetic signal.

## Data Availability Statement

The raw data supporting the conclusions of this article will be made available by the authors, without undue reservation.

## Author Contributions

TP, MW, and MZ designed the study and conducted field work and sampling. TP prepared the samples and performed xylem anatomical measurements with help from GA. MZ performed the genetic analyses. TP performed all statistical analysis with help from AA-R. TP wrote the manuscript with contributions from all authors.

## Conflict of Interest

The authors declare that the research was conducted in the absence of any commercial or financial relationships that could be construed as a potential conflict of interest.

## Footnotes

1 http://cfs.nrcan.gc.ca/projects/3/1

## References

[B1] Abrahamson I. (2015). *Fire Effects Information System: Picea glauca.* Available online at: https://www.feis-crs.org/feis/ (accessed on 31 January 2020)

[B2] Amoroso M. M. Daniels L. D. Baker P. J. Camarero J. J. (2017). *Dendroecology.* Cham: Springer International Publishing.

[B3] Apps M. J. Kurz W. A. Luxmoore R. J. Nilsson L. O. Sedjo R. A. Schmidt R. (1993). Boreal forests and tundra. *Water Air Soil Pollut.* 70 39–53. 10.1007/BF01104987

[B4] Arneth A. Harrison S. P. Zaehle S. Tsigaridis K. Menon S. Bartlein P. J. (2010). Terrestrial biogeochemical feedbacks in the climate system. *Nat. Geosci.* 3 525–532. 10.1038/ngeo905

[B5] Aroca R. (2012). *Plant Responses to Drought Stress.* Heidelberg: Springer.

[B6] Attree S. M. Dunstan D. I. Fowke L. C. (1991). *Trees III.* Heidelberg: Springer.

[B7] Avanzi C. Piermattei A. Piotti A. Büntgen U. Heer K. Opgenoorth L. (2018). Disentangling the effects of spatial proximity and genetic similarity on individual growth performances in Norway spruce natural populations. *Sci. Total Environ.* 650 493–504. 10.1016/j.scitotenv.2018.08.348 30199693

[B8] Björklund J. Seftigen K. Fonti P. Nievergelt D. von Arx G. (2020). Dendroclimatic potential of dendroanatomy in temperature-sensitive *Pinus sylvestris*. *Dendrochronologia* 60:125673 10.1016/j.dendro.2020.125673

[B9] Björklund J. Seftigen K. Schweingruber F. Fonti P. von Arx G. Bryukhanova M. V. (2017). Cell size and wall dimensions drive distinct variability of earlywood and latewood density in Northern Hemisphere conifers. *New Phytol.* 3 728–740. 10.1111/nph.14639 28636081

[B10] Burnham K. P. Anderson D. R. (2002). *Model Selection and Multimodel Inference: A Practical Information-Theoretic Approach.* New York, NY: Springer.

[B11] Burnham K. P. Anderson D. R. (2004). Multimodel inference: understanding AIC and BIC in model selection. *Sociol. Methods Res.* 33 261–304. 10.1177/0049124104268644

[B12] Carlquist S. (1980). Further concepts in ecological wood anatomy, with comments on recent work in wood anatomy and evolution. *Aliso* 9 499–553. 10.5642/aliso.19800904.02

[B13] Carrer M. (2011). Individualistic and time-varying tree-ring growth to climate sensitivity. *PLoS One* 6:e22813. 10.1371/journal.pone.0022813 21829523 PMC3145760

[B14] Carrer M. von Arx G. Castagneri D. Petit G. (2015). Distilling allometric and environmental information from time series of conduit size: the standardization issue and its relationship to tree hydraulic architecture. *Tree Physiol.* 35 27–33. 10.1093/treephys/tpu108 25576756

[B15] Cartenì F. Deslauriers A. Rossi S. Morin H. De Micco V. Mazzoleni S. (2018). The physiological mechanisms behind the earlywood-to-latewood transition: a process-based modeling approach. *Front. Plant Sci.* 9:1053. 10.3389/fpls.2018.01053 30079078 PMC6063077

[B16] Chambers J. M. Hastie T. (1992). *Statistical Models in S.* Pacific Grove, CA: Wadsworth & Brooks.

[B17] Charney N. D. Babst F. Poulter B. Record S. Trouet V. M. Frank D. (2016). Observed forest sensitivity to climate implies large changes in 21st century North American forest growth. *Ecol. Lett.* 19 1119–1128. 10.1111/ele.12650 27434040

[B18] Correia D. L. P. Bouchard M. Filotas É Raulier F. (2018). Disentangling the effect of drought on stand mortality and productivity in northern temperate and boreal forests. *J. Appl. Ecol.* 56 758–768. 10.1111/1365-2664.13305 29672967

[B19] Corriveau A. Beaulieu J. Daoust G. (1991). Heritability and genetic correlations of wood characters of Upper Ottawa Valley white spruce populations grown in Quebec. *For. Chron.* 67, 698–705. 10.5558/tfc67698-6

[B20] Denne M. P. (1989). Definition of latewood according to Mork (1928). *IAWA J.* 10 59–62. 10.1163/22941932-90001112

[B21] Domec J.-C. (2002). How do water transport and water storage differ in coniferous earlywood and latewood? *J. Exp. Bot.* 53 2369–2379. 10.1093/jxb/erf100 12432029

[B22] Eusemann P. Herzig P. Kieß M. Ahlgrimm S. Herrmann P. Wilmking M. (2014). Three microsatellite multiplex PCR assays allowing high resolution genotyping of white spruce, Picea glauca. *Silvae Genet.* 63 230–234. 10.1515/sg-2014-0029

[B23] Everitt B. S. (1999). The Cambridge dictionary of statistics. *J. Am. Stat. Assoc.* 94:657 10.2307/2670205

[B24] Gauthier S. Bernier P. Kuuluvainen T. Shvidenko A. Z. Schepaschenko D. G. (2015). Boreal forest health and global change. *Science* 349 819–822. 10.1126/science.aaa9092 26293953

[B25] Grey D. R. Mardia K. V. Kent J. T. Bibby J. M. (1981). Multivariate Analysis. *Math. Gaz.* 65:75 10.2307/3617970

[B26] Hacke U. G. Lachenbruch B. Pittermann J. Mayr S. Domec J.-C. Schulte P. J. (2015). “The hydraulic architecture of conifers,” in *Functional and Ecological Xylem Anatomy*, (Cham: Springer International Publishing).

[B27] Hansen M. C. DeFries R. S. Townshend J. R. G. Carroll M. Dimiceli C. Sohlberg R. A. (2003). Global percent tree cover at a spatial resolution of 500 meters: first results of the MODIS vegetation continuous fields algorithm. *Earth Interact.* 7 1–15. 10.1175/1087-35622003007<0001:GPTCAA<2.0.CO;2

[B28] Hansen M. C. Stehman S. V. Potapov P. V. (2010). Quantification of global gross forest cover loss. *Proc. Natl. Acad. Sci. U.S.A.* 107 8650–8655. 10.1073/pnas.0912668107 20421467 PMC2889354

[B29] Hassegawa M. Savard M. Lenz P. R. N. Duchateau E. Gélinas N. Bousquet J. (2019). White spruce wood quality for lumber products: priority traits and their enhancement through tree improvement. *For. An Int. J. For. Res.* 93 1–22. 10.1093/forestry/cpz050

[B30] Hill W. G. Goddard M. E. Visscher P. M. (2008). Data and theory point to mainly additive genetic variance for complex traits. *PLoS Genet.* 4:1–10. 10.1371/journal.pgen.1000008 18454194 PMC2265475

[B31] Hodgetts R. B. Aleksiuk M. A. Brown A. Clarke C. Macdonald E. Nadeem S. (2001). Development of microsatellite markers for white spruce (Picea glauca) and related species. *Theor. Appl. Genet.* 102 1252–1258. 10.1007/s00122-001-0546-0

[B32] Hughes M. K. Swetnam T. W. Diaz H. F. (2011). *Dendroclimatology*, eds Hughes M. K. Swetnam T. W. Diaz H. F. (Dordrecht: Springer Netherlands).

[B33] Hurvich C. M. Tsai C.-L. (1991). Bias of the corrected AIC criterion for underfitted regression and time series models. *Biometrika* 78 499–509. 10.1093/biomet/78.3.499

[B34] IPCC (2013). “Climate Change 2013: the physical science basis,” in *Contribution of Working Group I to the Fifth Assessment Report of the Intergovernmental Panel on Climate Change*, eds Stocker T. F. Qin D. Plattner G.-K. Tignor M. Allen S. K. Boschung J. (Cambridge: Cambridge University Press).

[B35] King G. M. Gugerli F. Fonti P. Frank D. C. (2013). Tree growth response along an elevational gradient: climate or genetics? *Oecologia* 173 1587–1600. 10.1007/s00442-013-2696-6 23771802

[B36] Klisz M. Ukalska J. Koprowski M. Tereba A. Puchałka R. Przybylski P. (2019). Effect of provenance and climate on intra-annual density fluctuations of Norway spruce Picea abies (L.) Karst. in Poland. *Agric. For. Meteorol.* 269–270 145–156. 10.1016/j.agrformet.2019.02.013

[B37] Klug W. S. Cummings M. R. Spencer C. A. Palladino M. A. (2006). in *Concepts of Genetics*, 9th Edn, ed. Wilbur B. (San Francisco, CA: Pearson Benjamin Cummings).

[B38] Kolb K. J. Sperry J. S. (1999). Transport constraints on water use by the Great Basin shrub, Artemisia tridentata. *Plant Cell Environ.* 22 925–935. 10.1046/j.1365-3040.1999.00458.x

[B39] Lange J. Carrer M. Pisaric M. F. J. Porter T. J. Seo J. W. Trouillier M. (2019). Moisture-driven shift in the climate sensitivity of white spruce xylem anatomical traits is coupled to large-scale oscillation patterns across northern treeline in northwest North America. *Glob. Chang. Biol.* 26 1842–1856. 10.1111/gcb.14947 31799729

[B40] Lenoir J. Gegout J. C. Marquet P. A. de Ruffray P. Brisse H. (2008). A significant upward shift in plant species optimum elevation during the 20th century. *Science* 320 1768–1771. 10.1126/science.1156831 18583610

[B41] Lenz P. Cloutier A. MacKay J. Beaulieu J. (2010). Genetic control of wood properties in Picea glauca — an analysis of trends with cambial age. *Can. J. For. Res.* 40 703–715. 10.1139/X10-014

[B42] Lenz P. MacKay J. Rainville A. Cloutier A. Beaulieu J. (2011). The influence of cambial age on breeding for wood properties in Picea glauca. *Tree Genet. Genomes* 7 641–653. 10.1007/s11295-011-0364-8

[B43] Little E. L. Viereck L. A. (1971). *Atlas of United States trees.* Washington, DC: U.S. Dept. of Agriculture, Forest Service.

[B44] Lloyd A. H. Fastie C. L. (2002). Spatial and temporal variability in the growth and climate response of treeline trees in Alaska. *Clim. Change* 52 481–509. 10.1023/a:1014278819094

[B45] McKenney D. W. Hutchinson M. F. Papadopol P. Lawrence K. Pedlar J. Campbell K. (2011). Customized spatial climate models for North America. *Bull. Am. Meteorol. Soc.* 92 1611–1622. 10.1175/2011bams3132.1

[B46] Merrill R. E. Mohn C. A. (1985). Heritability and genetic correlations for stem diameter and branch characteristics in white spruce. *Can. J. For. Res.* 15, 494–497. 10.1139/x85-081

[B47] Montpellier E. E. Soulé P. T. Knapp P. A. Shelly J. S. (2018). Divergent growth rates of alpine larch trees (Larix lyallii Parl.) in response to microenvironmental variability. *Arctic Antarct. Alp. Res.* 50 1–9. 10.1080/15230430.2017.1415626

[B48] Mvolo C. S. Koubaa A. Beaulieu J. Cloutier A. Defo M. Yemele M. C. (2019). Phenotypic correlations among growth and selected wood properties in white spruce (Picea glauca (Moench) Voss). *Forests* 10 1–17. 10.3390/f10070589

[B49] Nyquist W. E. Baker R. J. (1991). Estimation of heritability and prediction of selection response in plant populations. *CRC Crit. Rev. Plant Sci.* 10 235–322. 10.1080/07352689109382313

[B50] Pinheiro J. Bates D. DebRoy S. Sarkar D. R Core Team. (2020). *{nlme}**: Linear and Nonlinear Mixed Effects Models.* Available online at: https://cran.r-project.org/package=nlme (accessed June 4, 2020).

[B51] Prendin A. L. Petit G. Carrer M. Fonti P. Björklund J. Von Arx G. (2017). New research perspectives from a novel approach to quantify tracheid wall thickness. *Tree Physiol.* 37 1–8. 10.1093/treephys/tpx037 28379577

[B52] Puchi P. F. Castagneri D. Rossi S. Carrer M. (2020). Wood anatomical traits in black spruce reveal latent water constraints on the boreal forest. *Glob. Chang. Biol.* 26 1767–1777. 10.1111/gcb.14906 31692158

[B53] R Core Team (2019). *R: A Language and Environment for Statistical Computing.* Available online at: https://www.r-project.org/ (accessed July 31, 2019).

[B54] Rajora O. P. Rahman M. H. Dayanandan S. Mosseler A. (2001). Isolation, characterization, inheritance and linkage of microsatellite DNA markers in white spruce (Picea glauca) and their usefulness in other spruce species. *Mol. Gen. Genet.* 264 871–882. 10.1007/s004380000377 11254135

[B55] Rathgeber C. B. K. Cuny H. E. Fonti P. (2016). Biological basis of tree-ring formation: a crash course. *Front. Plant Sci.* 7:734. 10.3389/fpls.2016.00734 27303426 PMC4880555

[B56] Reich P. B. Sendall K. M. Stefanski A. Wei X. Rich R. L. Montgomery R. A. (2016). Boreal and temperate trees show strong acclimation of respiration to warming. *Nature* 531 633–636. 10.1038/nature17142 26982730

[B57] Schlichting C. (1986). The evolution of phenotypic plasticity in plants. *Annu. Rev. Ecol. Syst.* 17 667–693. 10.1146/annurev.ecolsys.17.1.667

[B58] Schnittler M. Eusemann P. (2010). Consequences of genotyping errors for estimation of clonality: a case study on Populus euphratica Oliv. (Salicaceae). *Evol. Ecol.* 24 1417–1432. 10.1007/s10682-010-9389-y

[B59] Sherriff R. L. Miller A. E. Muth K. Schriver M. Batzel R. (2017). Spruce growth responses to warming vary by ecoregion and ecosystem type near the forest-tundra boundary in south-west Alaska. *J. Biogeogr.* 44 1457–1468. 10.1111/jbi.12968

[B60] Soja A. J. Tchebakova N. M. French N. H. F. Flannigan M. D. Shugart H. H. Stocks B. J. (2007). Climate-induced boreal forest change: predictions versus current observations. *Glob. Planet Change* 56 274–296. 10.1016/j.gloplacha.2006.07.028

[B61] Stoltenberg S. F. (1997). Coming to terms with heritability. *Genetica* 99 89–96. 10.1007/BF02259512 9463077

[B62] Stone E. L. McKittrick R. C. (1976). On the layering of white spruce. *Tree Plant. Notes* 27:14.

[B63] Tagesson T. Schurgers G. Horion S. Ciais P. Tian F. Brandt M. (2020). Recent divergence in the contributions of tropical and boreal forests to the terrestrial carbon sink. *Nat. Ecol. Evol.* 4 202–209. 10.1038/s41559-019-1090-0 31988446

[B64] Tyree M. T. Zimmermann M. H. (2002). *Xylem Structure and the Ascent of Sap.* Berlin: Springer.

[B65] Visscher P. M. Hill W. G. Wray N. R. (2008). Heritability in the genomics era - Concepts and misconceptions. *Nat. Rev. Genet.* 9 255–266. 10.1038/nrg2322 18319743

[B66] von Arx G. Carrer M. (2014). Roxas -a new tool to build centuries-long tracheid-lumen chronologies in conifers. *Dendrochronologia* 32 290–293. 10.1016/j.dendro.2013.12.001

[B67] Wang Y. Vik J. O. Omholt S. W. Gjuvsland A. B. (2013). Effect of regulatory architecture on broad versus narrow sense heritability. *PLoS Comput. Biol.* 9:e1003053. 10.1371/journal.pcbi.1003053 23671414 PMC3649986

[B68] Wiedenhoeft A. C. (2012). *Handbook of Wood Chemistry and Wood Composites.* Boca Raton, FL: CRC Press.

[B69] Willson C. J. Jackson R. B. (2006). Xylem cavitation caused by drought and freezing stress in four co-occurring Juniperus species. *Physiol. Plant* 127 374–382. 10.1111/j.1399-3054.2006.00644.x

[B70] Wilmking M. Buras A. Eusemann P. Schnittler M. Trouillier M. Würth D. (2017). High frequency growth variability of White spruce clones does not differ from non-clonal trees at Alaskan treelines. *Dendrochronologia* 44 187–192. 10.1016/j.dendro.2017.05.005

[B71] Wilmking M. Juday G. P. (2005). Longitudinal variation of radial growth at Alaska’s northern treeline - recent changes and possible scenarios for the 21st century. *Glob. Planet Change* 47 282–300. 10.1016/j.gloplacha.2004.10.017

[B72] Wray N. R. Visscher P. M. (2008). Estimating trait heritability. *Nat. Educ.* 1:29.

[B73] Würth D. G. Eusemann P. Trouillier M. Buras A. Burger A. Wilmking M. (2018). Environment drives spatiotemporal patterns of clonality in white spruce (Picea glauca) in Alaska. *Can. J. For. Res.* 48 1577–1586. 10.1139/cjfr-2018-0234

[B74] Yeaman S. Hodgins K. A. Lotterhos K. E. Suren H. Nadeau S. Degner J. C. (2016). Convergent local adaptation to climate in distantly related conifers. *Science* 353 1431–1433. 10.1126/science.aaf7812 27708038

[B75] Ying C. C. Morgenstern E. K. (1979). Correlations of height growth and heritabilities at different ages in white spruce. *Silvae Genet.* 28.

