# Supplementary material for: Xylem Anatomical Variability in White Spruce at Treeline Is Largely Driven by Spatial Clustering
Source: Front Plant Sci. 2020 Oct 21;11:581378. doi: 10.3389/fpls.2020.581378 (PMC7609655; doi:10.3389/fpls.2020.581378)
Supplement: Supplementary file 1 [file Data_Sheet_1.docx]

# Supplementary Material


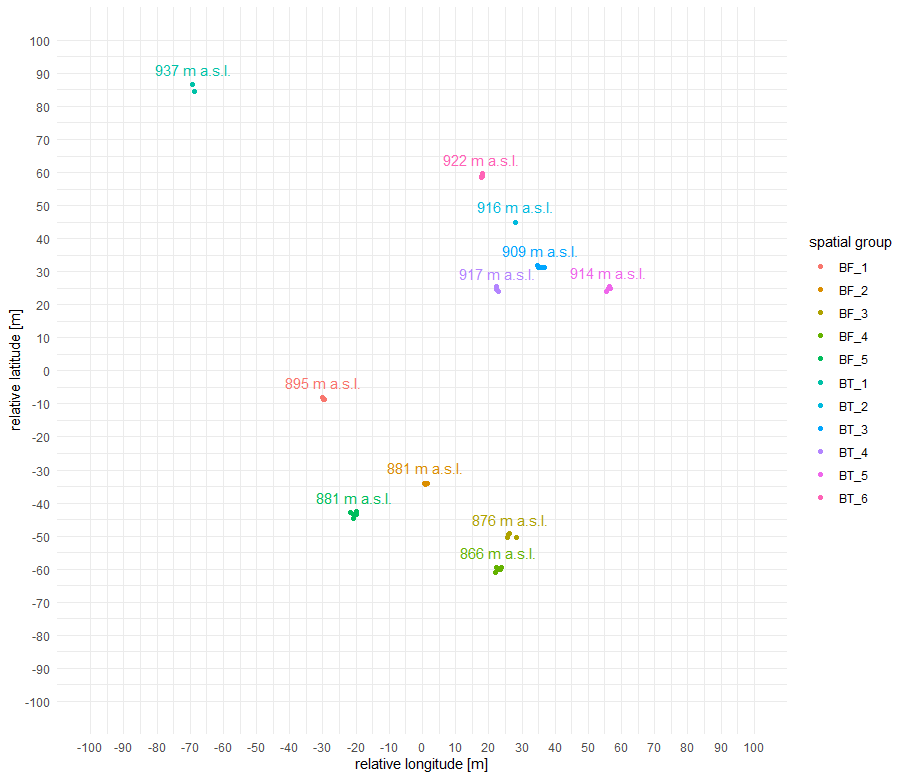


# Figure S1: Map of sampled trees. Dots represent the sampled trees on a relative coordinate system in metric units. Distances were measured using a Vertex Laser Geo (Haglöf, Sweden). Colors indicate the spatial group. For some groups not all trees can be represented, since the distance between the trees was smaller than the measuring error (ultra-sound accuracy: 1%). Numbers above the groups show the group mean elevation in meters above sea level, derived from GPS coordinates measured with the Vertex Laser Geo (GPS accuracy: 2.5m in open terrain).

#
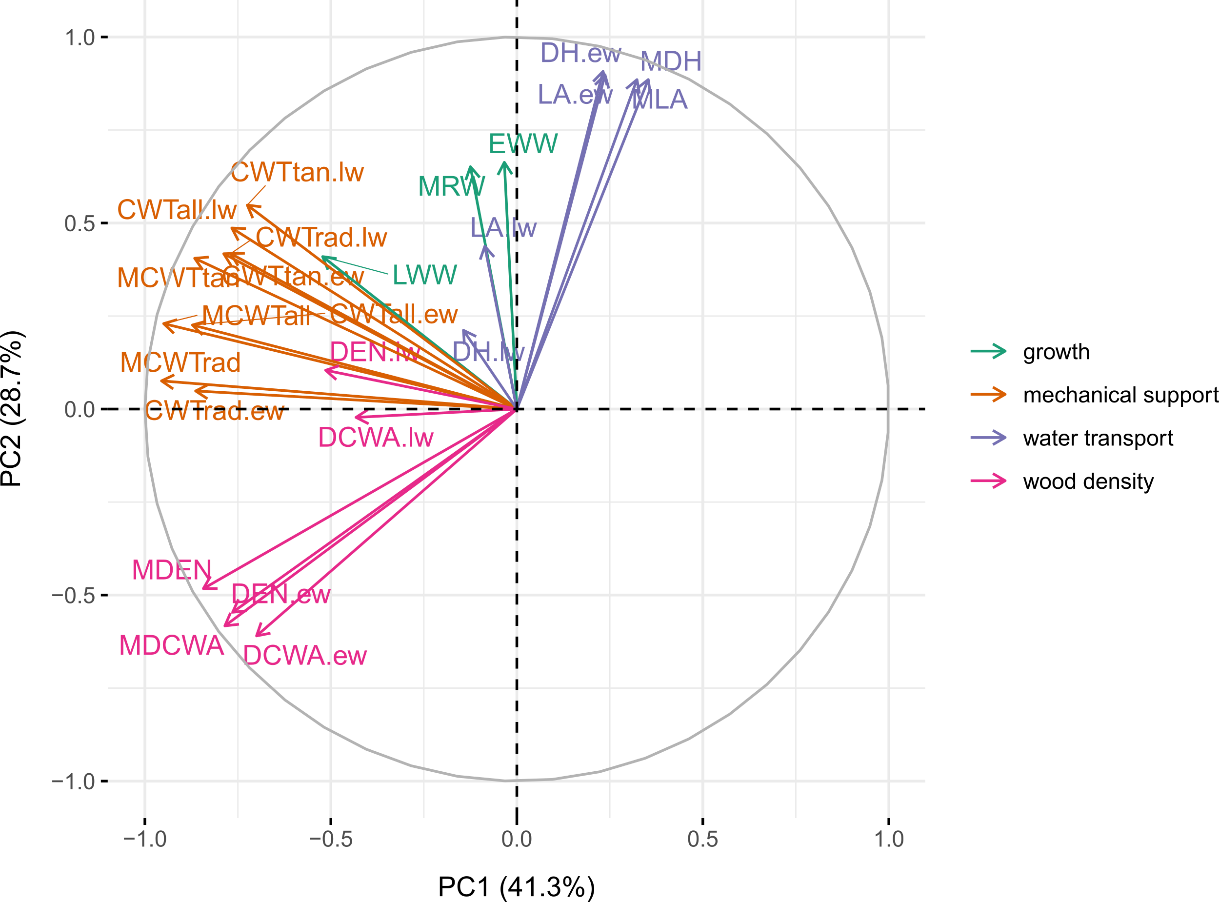


# Figure S2: Principal component analysis (PCA) of all measured growth and xylem anatomical traits. Colors indicate different trait groups (green = growth, orange = mechanical support, purple = water transport, pink = wood density).

Table S1: Metadata on sampled trees

| ID | coring direction | coring height (cm) | DBH (cm) | height (m) | clone_group | spatial_group | relative longitude (m) | relative latitude (m) | altitude (m a.s.l.) |
| --- | --- | --- | --- | --- | --- | --- | --- | --- | --- |
| 101b | N_S | 130 | 18 | 11.2 | BT_F | BT_4 | 22.3 | 25.7 | 917 |
| 102b | E_W | 140 | 13 | 8.40 | BT_F | BT_4 | 22.4 | 24.6 | 917 |
| 103.1b | NE_SW | 110 | 11 | 7.70 | BT_F | BT_4 | 22.9 | 23.9 | 917 |
| 103.2b | N_S | 115 | 9 | 4.55 | BT_F | BT_4 | 22.9 | 23.9 | 917 |
| 112b | E_W | 80 | 6.5 | 3.60 | BT_B | BT_5 | 55.3 | 24.1 | 914 |
| 114b | N_S | 110 | 14.5 | 6.20 | BT_B | BT_5 | 56.4 | 25.6 | 914 |
| 115b | E_W | 100 | 11.5 | 5.58 | BT_B | BT_5 | 56.1 | 24.8 | 914 |
| 116b | N_S | 120 | 8 | 3.70 | BT_B | BT_5 | 56.6 | 24.9 | 914 |
| 126b | NE_SW | 110 | 8 | 4.12 | BT_D | BT_6 | 17.9 | 58.8 | 922 |
| 128b | NW_SE | 110 | 9 | 4.55 | BT_D | BT_6 | 17.6 | 58.5 | 922 |
| 131b | N_S | 145 | 13 | 7.50 | BT_D | BT_6 | 17.9 | 59.1 | 922 |
| 47b | N_S | 85 | 12 | 7.50 | BT_D | BT_6 | 17.9 | 59.6 | 922 |
| 150b | N_S | 100 | 8 | 5.00 | BF_C | BF_1 | -29.6 | -8.7 | 895 |
| 151b | N_S | 95 | 12 | 5.50 | BF_C | BF_1 | -29.8 | -8.7 | 895 |
| 152b | E_W | 75 | 10 | 6.80 | 152b | BF_1 | -30.3 | -8.0 | 895 |
| 216b | N_S | 150 | 16 | 7.33 | 216b | BF_2 | 0.6 | -34.1 | 881 |
| 217b | S_N | 120 | 14 | 6.57 | 217b | BF_2 | 1.0 | -34.4 | 881 |
| 218b | W_E | 135 | 16.5 | 7.52 | 218b | BF_2 | 1.5 | -34.1 | 881 |
| 279b | NW_SE | 150 | 15.5 | 7.14 | BF_A | BF_3 | 28.4 | -50.4 | 876 |
| 281b | N_S | 160 | 13.5 | 6.37 | BF_A | BF_3 | 26.1 | -49.3 | 876 |
| 284b | NW_SE | 115 | 15 | 6.95 | BF_A | BF_3 | 25.5 | -50.3 | 876 |
| 285b | N_S | 115 | 11.5 | 5.58 | BF_A | BF_3 | 26.0 | -49.5 | 876 |
| 292b | NE_SW | 80 | 11.5 | 5.58 | BF_D | BF_4 | 23.7 | -59.6 | 866 |
| 293b | NW_SE | 120 | 10.5 | 5.17 | BF_D | BF_4 | 23.4 | -60.2 | 866 |
| 294b | N_S | 135 | 10.5 | 5.17 | BF_D | BF_4 | 23.3 | -60.0 | 866 |
| 295b | NE_SW | 110 | 13.5 | 6.37 | BF_D | BF_4 | 22.4 | -59.6 | 866 |
| 296b | W_E | 115 | 12 | 5.78 | BF_D | BF_4 | 21.9 | -60.9 | 866 |
| 32.1a | N_S | 110 | 14 | 5.20 | BT_E | BT_1 | -69.2 | 86.7 | 937 |
| 32.2a | N_S | 110 | 11.5 | 5.58 | 32.2a | BT_1 | -69.2 | 86.7 | 937 |
| 32.3b | E_W | 135 | 8.5 | 4.34 | BT_E | BT_1 | -69.2 | 86.7 | 937 |
| 32.4b | NW_SE | 120 | 10.5 | 5.17 | BT_E | BT_1 | -69.2 | 86.7 | 937 |
| 33b | S_N | 75 | 11 | 8.00 | 33b | BT_1 | -68.8 | 84.5 | 937 |
| 355b | NW_SE | 115 | 15 | 6.95 | 355b | BF_5 | -20.0 | -42.7 | 881 |
| 356b | N_S | 100 | 9 | 4.55 | 356b | BF_5 | -19.8 | -43.4 | 881 |
| 357b | NW_SE | 95 | 13 | 6.17 | 357b | BF_5 | -20.8 | -43.5 | 881 |
| 358a | N_S | 50 | 24 | 10.27 | 358a | BF_5 | -20.7 | -44.6 | 881 |
| 361b | W_E | 130 | 15 | 6.95 | 361b | BF_5 | -21.6 | -42.9 | 881 |
| 81.1b | W_E | 125 | 18 | 8.00 | BT_C | BT_2 | 28.1 | 44.8 | 916 |
| 81.2b | N_S | 120 | 18.5 | 8.27 | BT_C | BT_2 | 28.1 | 44.8 | 916 |
| 81.3a | S_N | 100 | 18.5 | 8.27 | BT_C | BT_2 | 28.1 | 44.8 | 916 |
| 81.4b | SE_NW | 135 | 10.5 | 5.17 | BT_C | BT_2 | 28.1 | 44.8 | 916 |
| 81.5b | SE_NW | 130 | 9.5 | 4.76 | BT_C | BT_2 | 28.1 | 44.8 | 916 |
| 95b | S_N | 120 | 10.5 | 4.80 | 95b | BT_3 | 36.6 | 31.4 | 909 |
| 96a | N_S | 135 | 11.5 | 5.80 | BT_A | BT_3 | 35.7 | 31.3 | 909 |
| 97b | N_S | 115 | 8.5 | 3.80 | BT_A | BT_3 | 35.6 | 31.4 | 909 |
| 98b | NE_SW | 115 | 11.5 | 5.80 | BT_A | BT_3 | 34.8 | 31.2 | 909 |
| 99b | E_W | 125 | 11.5 | 5.40 | BT_A | BT_3 | 34.6 | 32.0 | 909 |

**Table S2:** List of all measured traits.

| Abbreviation | Trait | Group |
| --- | --- | --- |
| TRW | Tree ring width | Growth |
| EWW | Earlywood width | Growth |
| LWW | Latewood width | Growth |
| MCWT | Mean cell wall thickness | Mechanical support |
| CWT.ew | Earlywood cell wall thickness | Mechanical support |
| CWT.lw | Latewood cell wall thickness | Mechanical support |
| MCWTrad | Mean radial cell wall thickness | Mechanical support |
| CWTrad.ew | Earlywood radial cell wall thickness | Mechanical support |
| CWTrad.lw | Latewood radial cell wall thickness | Mechanical support |
| MCWTtan | Mean tangential cell wall thickness | Mechanical support |
| CWTtan.ew | Earlywood tangential cell wall thickness | Mechanical support |
| CWTtan.lw | Latewood tangential cell wall thickness | Mechanical support |
| MDEN | Mean wood density based on cwt | Wood density |
| DEN.ew | Earlywood density based on cwt | Wood density |

| DEN.lw | Latewood density based on cwt | Wood density |
| --- | --- | --- |
| MDCWA | Mean wood density based on cell wall area | Wood density |
| DCWA.ew | Earlywood density based on cell wall area | Wood density |
| DCWA.lw | Latewood density based on cell wall area | Wood density |
| MLA | Mean lumen area | Water transport |
| LA.ew | Earlywood lumen area | Water transport |
| LA.lw | Latewood lumen area | Water transport |
| MDH | Mean hydraulic diameter | Water transport |
| DH.ew | Earlywood hydraulic diameter | Water transport |
| DH.lw | Latewood hydraulic diameter | Water transport |

Equation S1 $\sigma_{P}^{2} = \sigma_{G}^{2} + \bar{\sigma}_{p}^{2}$

Where $\bar{\sigma}_{p}^{2}$ is the mean clonal group trait variance, calculated as:

Equation S2 $\bar{\sigma}_{p}^{2}= \frac{\sum_{j=1}^{N} \sigma_{j}^{2}}{N}$

Where $\sigma_{j}^{2}$is the trait variance in group *j* and N is the number of clonal groups:

Equation S3 $\sigma_{j}^{2} = \frac{\sum_{i = 1}^{n_{j}} {(x_{ij} - \bar{x}_{j})}^{2}}{n_{j}-1}$

Where $x_{ij}$ is the mean of the investigated trait across the study years 2007-2017 in individual *i* of clonal group *j*, $\bar{x}_{j}$ is the mean of the investigated trait in clonal group *j* and n is the number of individuals in group *j*:

Equation S4 $\bar{x}_{j} = \frac{\sum_{i = 1}^{n_{j}} x_{ij}}{n_{j}}$

Equation S5 $\sigma_{G}^{2} = \frac{\sum_{j = 1}^{N} {(\bar{x}_{j} - \bar{x})}^{2}}{N-1}$

Where $\bar{x}$ is the trait total mean.
